# Exhaled breath condensate metabolomics reveals inflammation-associated metabolic alterations and metabolites with possible relevance to virulence-related processes in pulmonary cryptococcosis

**DOI:** 10.3389/fcimb.2026.1889007

**Published:** 2026-09-09

**Authors:** Yingjie Chen, Qiusheng Yang, Lijuan Chen, Shuo Wei

**Affiliations:** 1 Shengli Clinical Medical College of Fujian Medical University, Fujian Medical University, Fuzhou, China; 2 Department of Infectious Diseases, Fuzhou University Affiliated Provincial Hospital, Fujian Medical University, Fuzhou, Fujian, China

**Keywords:** exhaled breath condensate, inflammation, lipid metabolism, liquid chromatography-mass spectrometry, metabolomics, pulmonary cryptococcosis

## Abstract

**Introduction:**

To analyze metabolic alterations associated with pulmonary cryptococcosis (PC) using metabolomic profiling of exhaled breath condensate (EBC) and investigate metabolic differences between fungal infections through comparison with patients with pulmonary aspergillosis (PA).

**Methods:**

We enrolled 32 patients with PC, 16 patients with PA, and 14 healthy controls (NC) between September 2022 and September 2025. EBC samples were analyzed using ultra-high-performance liquid chromatography–tandem mass spectrometry (UHPLC–MS/MS). Differential metabolites were identified using t-test, fold-change and PLS-DA. Discriminatory ability was evaluated using receiver operating characteristic (ROC) curves, and potential biological significance was explored through correlation analysis and KEGG pathway.

**Results:**

Forty-five differential metabolites were putatively annotated in the PC versus NC comparison, with 24 remaining significant after FDR correction (q < 0.05). Pathway analysis nominally implicated glycerophospholipid and amino acid metabolism pathways, suggesting that dysregulated lipid metabolism may contribute to the pathogenesis and progression of PC. ROC analysis in this single-center discovery cohort, without independent validation, revealed that alpha-ionone (AUC = 0.8482) and N,N-dimethylsphingosine (AUC = 0.8348) exhibited favorable preliminary discriminatory potential, reflecting distinct metabolic alterations associated with PC. Phenethylamine and phytosphingosine showed moderate preliminary discriminatory potential (AUC = 0.7679 and 0.7567, respectively), and their altered levels raise the possibility of an association with Cryptococcus virulence-related processes. Phytosphingosine was positively correlated with interleukin-6 (IL-6) (r = 0.73, raw P = 0.040), although not after FDR correction (q = 0.423). PC versus PA comparison putatively annotated eight differential metabolites (raw P < 0.05), none remaining significant after FDR correction (q = 0.535); these metabolites were nominally implicated in glycerophospholipid metabolism and polyunsaturated fatty acid-related pathways. Beta-ionone, butaxamine, and cimiracemoside D exhibited gradient trends across the three groups, while cimiracemoside D was positively correlated with erythrocyte sedimentation rate (ESR) (ρ = 0.48, raw P = 0.022), although not after FDR correction (q = 0.423), suggesting metabolic differences between fungal infections may reflect differences in inflammatory responses.

**Discussion:**

EBC metabolomic analysis revealed significant inflammation-related and lipid metabolism-related alterations in patients with PC versus healthy controls, while comparisons with PA suggested that these metabolic changes mainly reflected shared host responses rather than pathogen-specific features.

## 1 Introduction

Pulmonary cryptococcosis (PC) is the common clinical manifestation of *Cryptococcus* infections. Traditionally, this disease has been considered to occur predominantly in immunocompromised individuals (May et al., 2016); however, recent epidemiological data indicate an increasing proportion of immunocompetent individuals in China (Hou et al., 2019), while outbreaks of *Cryptococcus gattii* have shown that similar disease can affect otherwise healthy people in other temperate regions (Bielska and May, 2016). This shift complicates early disease recognition, as the clinical presentation is often nonspecific and some patients present with an insidious onset that is easily overlooked (Bielska and May, 2016).

Despite advances in diagnostic techniques, significant challenges remain in the clinical diagnosis of pulmonary cryptococcosis. Conventional methods, including fungal culture, histopathological examination, and detection of cryptococcal antigen (CrAg), have limitations. Culture methods are time-consuming and exhibit a low positivity rate (Setianingrum et al., 2019), with sensitivity declining further after the initiation of antifungal therapy. Although histopathological examination is considered a diagnostic reference standard, its invasive nature renders it unsuitable for repeated assessments. Serum CrAg testing is straightforward to perform, but its sensitivity is reduced in patients with localized lesions or immunocompetent individuals, increasing the risk of false-negative results (Zhu et al., 2018). The sensitivity and specificity of CrAg detection in bronchoalveolar lavage fluid (BALF) are 88.1% and 99.6%, respectively, showing high diagnostic accuracy for pulmonary cryptococcosis in HIV-negative patients (Chen et al., 2026). However, BALF collection is an invasive procedure, which may limit sample collection in patients who cannot tolerate the procedure. Furthermore, the diverse radiological presentations of PC overlap with those of other diseases such as lung cancer and pulmonary tuberculosis (Zhang et al., 2020). Some imaging signs can be shared between PC and pulmonary aspergillosis (PA), thereby increasing the difficulty of differential diagnosis (Alexander et al., 2021).

*Cryptococcus* primarily enters the human body through the inhalation of desiccated yeast cells or basidiospores from the environment, with the lungs serving as the initial site of colonization and infection. This pathogen employs various virulence factors, including polysaccharide capsules, melanin, extracellular vesicles, and titan cell formation, to survive and proliferate within the host. These mechanisms collectively contribute to immune evasion and persistent infection, thereby forming a complex pathogenic network (Bielska and May, 2016). Respiratory tract alterations associated with these host–pathogen interactions can potentially be captured in respiratory specimens.

Exhaled breath condensate (EBC), a noninvasive sample derived from the airway lining fluid, has garnered increasing attention. EBC contains volatile organic compounds and small amounts of nonvolatile components, such as metabolites, proteins, nucleic acids, and inflammatory mediators (Davis and Montpetit, 2018), which may reflect the local biochemical state of the respiratory tract. In recent years, metabolomic studies based on EBC have been increasingly applied in the field of respiratory diseases. Because EBC can reflect local pulmonary inflammatory and metabolic changes, it has been used to screen for potential diagnostic biomarkers in conditions such as pneumonia, pulmonary fibrosis, chronic obstructive pulmonary disease, and lung cancer, showing promising potential (Li et al., 2022; Chow et al., 2012; Freund et al., 2024; Wu et al., 2021).

EBC metabolomics has been explored most extensively in PA. In our previous study, UHPLC-HRMS profiling of EBC identified a five-metabolite panel—Asperpyrone C, Kotanin, Terphenyllin, Terrelumamide B, and Cyclotryprostatin D—that distinguished PA from controls with 100% sensitivity and 89.6% specificity (Wei et al., 2022). Breath volatile organic compound (VOC) studies have also reported strong diagnostic performance: Koo et al. (2014) detected a four-metabolite signature (three sesquiterpenes and a terpenoid ketone) that discriminated invasive aspergillosis from other pneumonias with 94% sensitivity and 93% specificity (Koo et al., 2014), whereas Li et al. (2021) identified a VOC panel separating chronic PA from community-acquired pneumonia with over 95% sensitivity and specificity (Li et al., 2021).

Whether comparable pathogen-specific signatures can be identified in other fungal infections remains unclear. In oral candidiasis, Hertel et al. (2018) observed breath VOCs alterations after antifungal therapy, yet no *Candida*-specific marker was identified; conventional microbial testing remained necessary for initial diagnosis (Hertel et al., 2018). Similarly, Bhimji et al. (2018) investigated whether galactomannan, an *Aspergillus* cell wall component, could be measured in EBC as a surrogate for BALF testing, but no correlation was observed, limiting its standalone utility (Bhimji et al., 2018).

Collectively, these studies demonstrate that breath and EBC can capture pathogen-specific metabolites and disease-associated volatile compounds in certain fungal infections, though the yield depends heavily on the analytical platform and the pathogen involved. Most existing work has focused on detecting pathogen-derived secondary metabolites; host inflammatory and lipid metabolic responses reflected in EBC remain poorly characterized across different fungal infections.

However, studies investigating EBC metabolomics in PC remain limited. A previous study established an *in vitro* model of *Cryptococcus*-infected alveolar epithelial cells and analyzed the supernatant using gas chromatography–mass spectrometry to identify characteristic metabolites across different phases of infection (Liew et al., 2016). However, that study was based on an *in vitro* alveolar epithelial cell model and focused on metabolic profiles across infection phases without comparison with healthy controls or other fungal infections.

Moreover, metabolic differences between distinct pulmonary fungal infections have not been adequately investigated. Pulmonary aspergillosis (PA) and PC share overlapping clinical manifestations and radiological characteristics; however, their host inflammatory responses and pathogenic mechanisms differ. Whether these differences are reflected in the EBC metabolome remains unclear.

**Figure 1:**
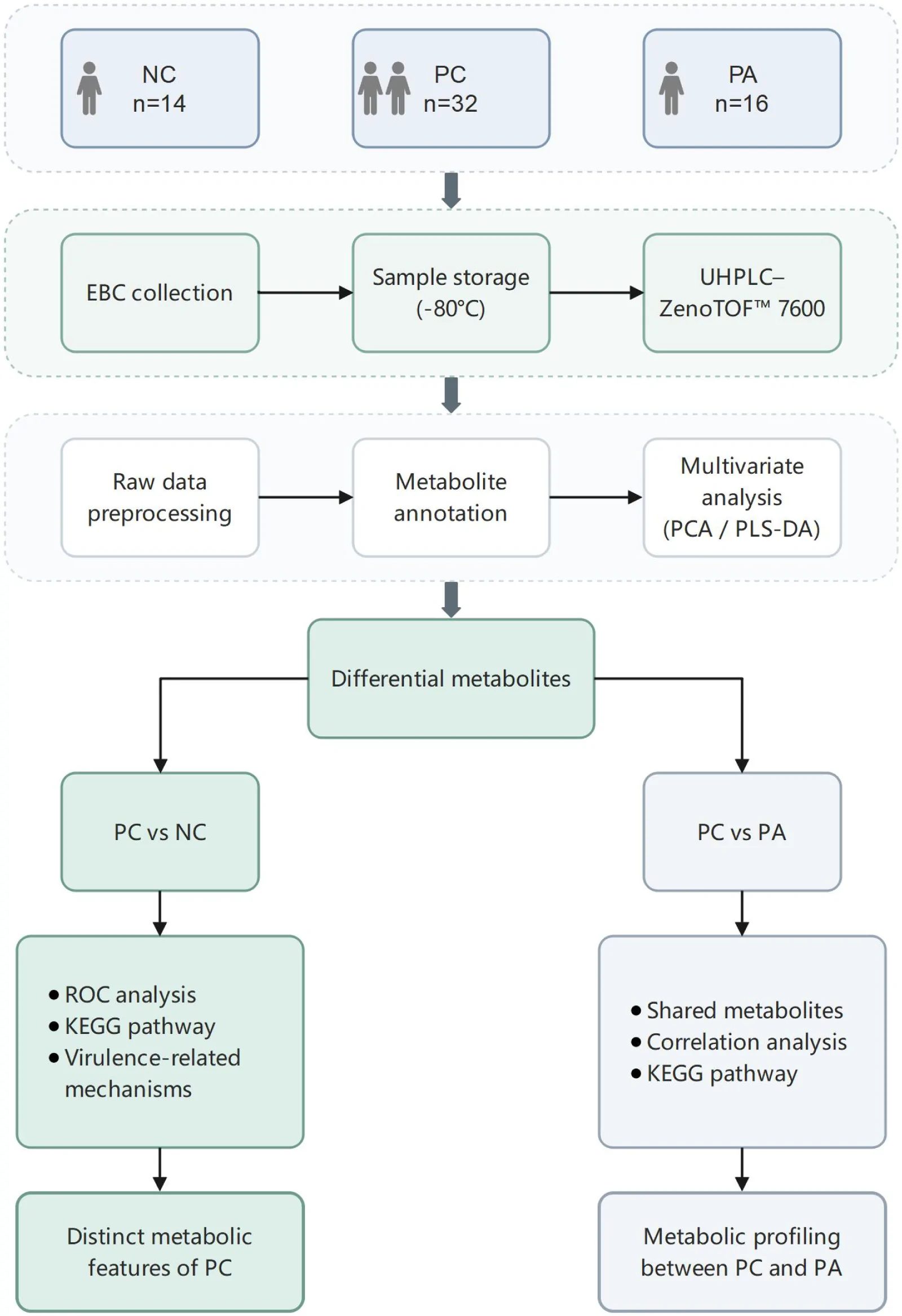
Study design and workflow. NC, healthy controls; PC, pulmonary cryptococcosis; PA, pulmonary aspergillosis.

In the present study, we employed an untargeted metabolomics approach to analyze EBC samples from patients with PC, patients with PA, and healthy individuals with the following objectives: (1) to characterize the EBC metabolic features of patients with PC compared with healthy controls; and (2) to compare metabolic differences between PC and PA and elucidate the associated metabolic pathways. A technical roadmap for this study is shown in **Figure 1**.

## 2 Materials and methods

### 2.1 Ethics

The study protocol was approved by the Ethics Committee of Fuzhou University Affiliated Provincial Hospital (Approval No.: K2022-05-004). Written informed consent was obtained from all participants.

### 2.2 Patients

This was a single-center prospective study. A total of 32 patients diagnosed with pulmonary cryptococcosis (PC group) at Fuzhou University Affiliated Provincial Hospital, Fujian Medical University between September 2022 and September 2025 were enrolled. Sixteen patients with pulmonary aspergillosis (PA group) and 14 healthy controls (NC group) were included. Pulmonary cryptococcosis was diagnosed according to the revised and updated consensus definitions for invasive fungal disease proposed by the European Organization for Research and Treatment of Cancer and the Mycoses Study Group Education and Research Consortium (EORTC/MSGERC) in 2019 (Donnelly et al., 2020). Inclusion criteria were as follows: presence of clinical symptoms such as cough, sputum production, chest pain, and low-grade fever; imaging findings suggestive of PC; and fulfillment of at least one of the following conditions: (i) positive serum or cerebrospinal fluid CrAg test; (ii) confirmation of *Cryptococcus* in lung tissue via histopathological biopsy (microscopic examination and special stains), culture, or next-generation sequencing; (iii) isolation of *Cryptococcus* species from culture of BALF, sputum, or pleural effusion; (iv) positive next-generation sequencing result from BALF; (v) positive BALF CrAg test. All included patients met the criteria for proven or probable diagnosis. The diagnosis of pulmonary aspergillosis also followed the EORTC/MSGERC criteria (Donnelly et al., 2020). Healthy volunteers had no history of acute or chronic respiratory diseases. Exclusion criteria were as follows: 1. Individuals unable to cooperate with EBC collection or those with severe respiratory insufficiency preventing completion of the expiratory sampling procedure; 2. Co-infection with other pathogens.

The 16 patients with pulmonary aspergillosis and 14 healthy controls recruited in the present study do not overlap with subjects in our previously published 2022 exhaled breath condensate metabolomics study of pulmonary aspergillosis.

### 2.3 Materials

Methanol, acetonitrile, and water were purchased from Fisher Chemical (Fair Lawn, NJ, USA). Formic acid and ammonium formate were obtained from Merck (Darmstadt, Germany). 2-Chloro-L-phenylalanine (internal standard, ≥98% purity) was purchased from Aladdin (Shanghai, China). EBC samples were collected using an RTube device (Respiratory Research, Austin, TX, USA). Chromatographic separation was performed on a Waters HSS T3 column (100 mm × 2.1 mm i.d., 1.8 µm; Waters Corporation, Milford, MA, USA) using an LC-40 UHPLC system (Shimadzu, Kyoto, Japan) coupled to a ZenoTOF™ 7600 high-resolution time-of-flight (HRTOF) mass spectrometer with a Turbo V™ electrospray ionization (ESI) source (AB SCIEX, Framingham, MA, USA). Low-temperature ultrasonic extraction was performed in an ultrasonic bath (Model SB-120DT, SCIENTZ, Ningbo, China). Centrifugation was performed using a refrigerated microcentrifuge (Model 5424R, Eppendorf, Hamburg, Germany). Vacuum drying was performed using a vacuum centrifugal concentrator (JX-ZLN-B, Jingxin, Shanghai, China).

### 2.4 EBC collection and processing

Sample preprocessing, LC–MS/MS analysis, metabolomic data processing, and statistical analyses for differential metabolite screening were performed at the Instrumental Analysis and Testing Center, Fujian Agriculture and Forestry University, Fuzhou, China. EBC samples were collected using a commercial device (RTube; Respiratory Research, USA). Participants refrained from eating and drinking for one hour prior to collection. They breathed quietly into the device for approximately 10 min to obtain an adequate volume of condensate (approximately 700–2500 μL). All samples were immediately stored at −80 °C until analysis.

Prior to processing, the samples were thawed slowly at 4 °C. A 200 μL aliquot of each sample was mixed with 800 μL of precooled methanol–acetonitrile–water (2:2:1, v/v). After vortexing for 10 s, the mixture was subjected to ultrasonic extraction at 6 °C for 30 min, incubated at −20 °C for 10 min, and then centrifuged at 14,000 × g at 4 °C for 20 min. The supernatant was collected and dried under vacuum. The dried residue was reconstituted in 200 μL of 50% methanol containing 4 ppm 2-chloro-L-phenylalanine as an internal standard. Following centrifugation at 14,000 × g at 4 °C for 10 min, the supernatant was used for LC-MS/MS analysis. Quality control (QC) samples were prepared by pooling equal volumes of all individual samples and processing them using the identical procedure described above to monitor instrument performance and analytical stability.

### 2.5 Ultra-high performance liquid chromatography coupled with high-resolution time-of-flight mass spectrometry

Metabolomic profiling was performed using a UHPLC system coupled to a ZenoTOF™ 7600 mass spectrometer. Chromatographic separation was achieved on a Waters HSS T3 column (100 mm × 2.1 mm, 1.8 μm). In positive ion mode, mobile phase A consisted of 0.1% formic acid in water, whereas in negative ion mode it consisted of 10 mM ammonium formate. Mobile phase B consisted of acetonitrile. A gradient elution program was used: 0−0.5 min, 95% A and 5% B; 0.5−7 min, 95−10% A and 5−90% B; 7−9 min, 10% A and 90% B; 9−10 min, 10−95% A and 90−5% B; 10−12 min, 95% A and 5% B. The column temperature was maintained at 40 °C, and the flow rate was 0.25 mL/min. Mass spectrometry data were acquired in positive and negative ion modes over a mass range of 100–1200 m/z. The ion source parameters were set as follows: curtain gas, 35 psi; source gas 1, 50 psi; source gas 2, 55 psi; source temperature, 450 °C; ion spray voltage, 5500 V in positive mode and −4500 V in negative mode; and declustering potential, 60 V. Tandem mass spectrometry (MS/MS) data were acquired in data-dependent acquisition (DDA) mode. The maximum number of candidate ions was set to 10, with an intensity threshold of 50 cps. Stepped collision energies of 25, 35, and 45 eV were used for MS/MS fragmentation.

### 2.6 Patient data analysis and metabolite identification

Continuous variables were tested for normality. Variables following a normal distribution are expressed as mean ± standard deviation and were compared using an unpaired t-test. Variables with a non-normal distribution are expressed as median (interquartile range) and were compared using the Mann–Whitney U test. Categorical variables are presented as counts (percentages) and were analyzed using the chi-square test or Fisher’s exact test. Statistical significance was set at P-value < 0.05.

Raw data were processed using MExplorer Ultimate software, including peak extraction, peak alignment, and normalization. To ensure data quality, metabolic features were filtered using the 80% rule, and features with a relative standard deviation (RSD) > 30% in QC samples were excluded.

Metabolites were putatively annotated by matching MS and MS/MS spectra against the HMDB, METLIN, MoNA, and GNPS databases.

Differential metabolites were screened using univariate and multivariate analyses. Univariate analysis employed Welch's t-test and fold change (FC) analysis, with thresholds set at P < 0.05, FC > 1.5, or FC < 0.67. Multivariate analysis was performed using principal component analysis (PCA) and partial least squares discriminant analysis (PLS-DA). Metabolites with variable importance in projection (VIP) score > 1 were considered significant. The stability of the PLS-DA model was evaluated using permutation tests. Multiple testing was controlled using the Benjamini–Hochberg FDR procedure for differential metabolite screening, pathway enrichment, and correlation analyses; adjusted q values are reported with raw P values.

Differential metabolites were subsequently submitted to the KEGG database for pathway enrichment analysis.

ROC curves were generated to evaluate discriminatory abilities, and area under the curve (AUC), sensitivity, and specificity were calculated. Correlations between five differential metabolites (beta-ionone, butaxamine, cimiracemoside D, phytosphingosine, and phenethylamine) and inflammatory markers including C-reactive protein (CRP), erythrocyte sedimentation rate (ESR), interleukin-6 (IL-6), ferritin, and procalcitonin (PCT), were assessed using Pearson’s or Spearman’s correlation analyses, as appropriate.

Clinical characteristics and correlation analyses were performed using GraphPad Prism 10 software. Statistical significance was set at P-value < 0.05.

## 3 Results

### 3.1 Clinical characteristics of the study participants

A total of 32 patients in the PC group, 16 in the PA group, and 14 in the NC group were enrolled. There were no statistically significant differences among the three groups in terms of age, sex, smoking history, or distribution of risk factors (P > 0.05), indicating comparable baseline characteristics across the groups (**Table 1**). The risk factors were defined according to the Global Guidelines for the Diagnosis and Management of Cryptococcosis, an initiative of the ECMM and ISHAM in cooperation with the ASM (Chang et al., 2024).

**Table 1:** Analysis of general clinical characteristics of the study participants.

Characteristic	PC group	PA group	P value (PC vs PA)	NC group	P value (PC vs NC)
Number (n)	32	16		14	
Age (years), mean ± SD	48.5 ± 17.1	58.4 ± 16.7	0.067	39.9 ± 11.4	0.096
Sex, n (%)			0.217		0.371
Male	16 (50.0)	11 (68.8)		5 (35.7)	
Female	16 (50.0)	5 (31.3)		9 (64.3)	
Smoking history, n (%)			0.090		0.413
Yes	6 (18.8)	7 (43.8)		1 (7.1)	
No	26 (81.3)	9 (56.3)		13 (92.9)	
Risk factors, n (%)	8 (25.0)	5 (31.3)	0.735	0 (0.0)	0.085

According to the Global Guidelines for the Diagnosis and Management of Cryptococcosis, a Joint initiative of ECMM, ISHAM, and ASM, risk factors for cryptococcal infection include human immunodeficiency virus (HIV) infection, solid organ transplantation, hematologic malignancies, liver cirrhosis, rheumatoid arthritis, sarcoidosis, use of immunosuppressive agents (e.g., glucocorticoids, ibrutinib, Janus kinase inhibitors), idiopathic CD4 lymphocytopenia, and rare primary immunodeficiency diseases.

In addition, statistical analysis of the sensitivity of different diagnostic methods for PC revealed that the serum CrAg test exhibited a high detection rate, whereas the positive rates for sputum and blood cultures were relatively low (**Table 2**).

**Table 2:** Sensitivity of different diagnostic methods for PC.

Diagnostic method	Positive cases/total tested	Sensitivity (%)	Sensitivity (%)	Specificity(%)
Serum CrAg test	21/24	87.5%	72.7%-90.5%^[1-4]^	98.7%-99.6%^[1-4]^
Blood culture	1/14	7.1%	3%^[1]^-4.5%^[6]^	100%^[1]^
Sputum culture	0/8	0.0%	11.1%^[7]^	–
Sputum cryptococcal DNA PCR assay	2/5	40.0%	–	–
BALF				
CrAg test	2/2	100.0%	88.1%-93.9%^[1,3-4]^	99.6%-100%^[1,3-4]^
Next-generation sequencing (NGS)	5/6	83.3%	85.71%-90.9%^[8]^	100.00%
Lung tissue histopathology	6/8	75.0%	86.3%^[5]^	100%^[5]^
Lung tissue culture	1/4	25.0%	47.1%^[5]^	100%^[5]^
Lung tissue NGS	3/5	60.0%	66.7%^[8]^	–

Sensitivity was calculated as positive cases/total tested cases ×100%.

1 (Zhu et al., 2022); 2 (Huang et al., 2026); 3 (Chen et al., 2026); 4 (Zeng et al., 2021); 5 (Liu et al., 2024); 6 (Singh et al., 2008); 7 (Huang et al., 2026); 8 (Huang et al., 2024).

### 3.2 Metabolic differences between PC and NC

**Figure 2:**
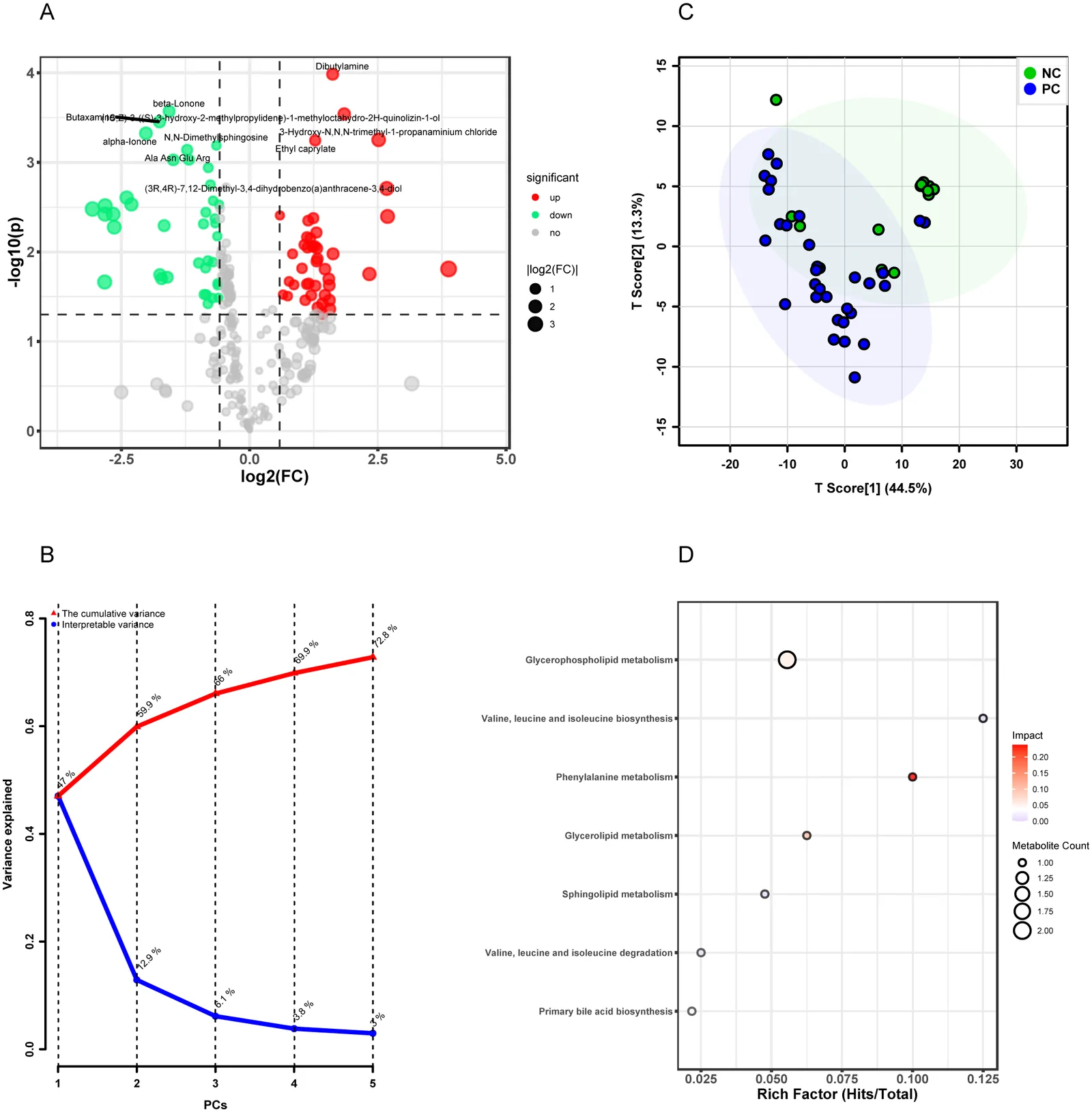
Metabolomic differences and pathway enrichment analysis between the PC and NC groups. **(A)** Volcano plot illustrating differential metabolites between the two groups, with red indicating upregulated metabolites, green indicating downregulated metabolites, and gray indicating non-significant metabolites. **(B)** Variance explained by principal components. The two lines represent the percentage of variance explained by individual principal components and the cumulative percentage, respectively. The horizontal axis shows the principal component index, and the vertical axis shows the percentage of variance explained. **(C)** Partial least squares discriminant analysis (PLS-DA) score plot showing separation between the PC and NC groups. **(D)** Bubble plot showing KEGG pathway enrichment analysis of differential metabolites. Bubble size represents the number of matched metabolites, and color intensity reflects pathway impact value. Raw P values are shown; no pathway remained significant after FDR correction (see **Table 4**). NC, healthy controls; PC, pulmonary cryptococcosis.

Based on untargeted metabolomic analysis, 263 metabolites were putatively annotated. The results of the univariate analysis were visualized using a volcano plot (**Figure 2A**). Principal component analysis (PCA) indicated that the first two principal components explained 59.9% of the total variance, and the cumulative proportion reached 72.8% for the first five components (**Figure 2B**). Further analysis using partial least squares discriminant analysis (PLS-DA) enabled clear discrimination between the two groups (**Figure 2C**), suggesting systemic metabolic alterations in patients with PC. Permutation testing indicated that the intercepts were within acceptable limits of the model (R²X_0_ = 0.36, Q²X_0_ = –0.35).

Combining univariate and multivariate analyses with screening criteria of P < 0.05, VIP > 1, and FC > 1.5 or FC < 0.67, 45 differential metabolites were identified (**Table 3**), of which 24 remained significant after FDR correction (q < 0.05). Sixteen of the 45 were lipids and lipid-like molecules. According to the Metabolomics Standards Initiative (MSI) criteria (Sumner et al., 2007), all differential metabolites reported in this study were putatively annotated at confidence level 2.

**Table 3:** Differential metabolites between the PC and NC groups.

No.	Compounds	log_2_(FC)	P (t-test)	FDR	VIP
1	3-Hydroxy-N,N,N-trimethyl-1-propanaminium chloride	2.5162	0.001	0.021	1.28
2	Epsilon caprolactam	0.6517	0.030	0.077	1.40
3	Dibutylamine	1.6187	< 0.001	0.021	1.25
4	3-Methylbutyl angelate	-1.6668	0.005	0.039	1.09
5	3-Acrylamidopropyl trimethylammonium	0.59062	0.004	0.037	1.43
6	Ethyl caprylate	1.2783	0.001	0.021	1.43
7	beta-ionone	-1.5696	< 0.001	0.021	1.13
8	alpha-Ionone	-2.0223	0.001	0.021	1.23
9	N-Methyldodecylamine	-1.4887	0.001	0.022	1.08
10	(E)-8-methyl-5-(pent-2-en-4-yn-1-yl)-1,2,3,5,8,8a-hexahydroindolizine	-2.3971	0.003	0.037	1.26
11	Hydroxyphenylacetylglycine	0.7695	0.021	0.070	1.38
12	Pentaethylene glycol	-2.8264	0.022	0.070	1.19
13	Phenethylamine	-2.6621	0.004	0.037	1.20
14	(3aR,8R,8aR,9aR)-8-hydroxy-8a-methyl-3,5-dimethylidene-3a,4,4a,6,7,8,9,9a-octahydrobenzo[f][1]benzofuran-2-one	1.0217	0.015	0.064	1.11
15	9a-hydroxy-3,4a,5-trimethyl-2H,4H,4aH,5H,6H,7H,8H,8aH,9H,9aH-naphtho[2,3-b]furan-2-one	-1.1829	0.001	0.022	1.02
16	Trans-Hexa-dec-2-enoic acid	-0.99226	0.013	0.062	1.15
17	3-Methyl-2-Oxovalerate	1.1422	0.005	0.038	1.07
18	Imazapyr	-1.7504	0.018	0.067	1.15
19	Butaxamine	-1.7571	< 0.001	0.021	1.16
20	SPB 16:1;2O	-2.3052	0.003	0.037	1.18
21	Lauryldiethanolamine	1.0663	0.008	0.053	1.33
22	C16 Sphinganine	-3.0643	0.003	0.037	1.20
23	9(Z),11(E)-Conjugated Linoleic Acid	-0.85845	0.015	0.064	1.33
24	(3R,4R)-7,12-Dimethyl-3,4-dihydrobenzo(a)anthracene-3,4-diol	2.6741	0.002	0.034	1.17
25	Phytosphingosine	-2.6399	0.005	0.040	1.19
26	Denatonium (RT 5.94 min)	-2.8192	0.004	0.037	1.19
27	Denatonium (RT 6.63 min)	1.1173	0.007	0.047	1.45
28	N,N-Dimethylsphingosine	-0.6486	0.001	0.021	1.12
29	Piperonyl butoxide	-0.8859	0.030	0.077	1.74
30	Bursopoietin	1.1155	0.023	0.070	1.13
31	3-(benzo[d][1,3]dioxol-5-yl)-6-ethyl-7-isopropoxy-2-methyl-4H-chromen-4-one	-2.8176	0.003	0.037	1.21
32	NCGC00385130-01_C16H24O8_Hexopyranoside, 2-hydroxy-2-(4-methoxyphenyl)-1-methylethyl	0.7421	0.031	0.077	1.35
33	LysoPA(0:0/18:0)	1.2416	0.004	0.037	1.30
34	MG(0:0/22:0/0:0)	-1.6060	0.019	0.067	1.33
35	Decaethylene glycol	0.83716	0.011	0.058	1.21
36	(3R,5S,7R,9S,10S,12S,13R,14S,17R)-17-((2R)-7-hydroxy-6-methylheptan-2-yl)-10,13-dimethylhexadecahydro-1H-cyclopenta[a]phenanthrene-3,7,12-triol	-1.7170	0.020	0.069	1.38
37	Bis(2,2,6,6-tetramethyl-4-piperidyl) sebacate	0.8635	0.023	0.070	1.36
38	Ala Asn Glu Arg	-1.2183	0.001	0.021	1.03
39	Asn Ala Leu Ala His	1.3023	0.006	0.044	1.04
40	PG 4:0_17:2	1.1293	0.009	0.053	1.13
41	His Arg Lys Glu	1.2197	0.009	0.053	1.10
42	Phe Tyr Lys Arg	1.2714	0.009	0.053	1.11
43	PG 6:0_19:2	1.1518	0.023	0.070	1.05
44	Cimiracemoside D	1.4690	0.016	0.064	1.04
45	PIP(PGJ2/20:4(5Z,8Z,11Z,14Z))	2.3357	0.018	0.067	1.06

log2(FC) represents log2 fold change; P (t-test) indicates P value from Welch's t-test; VIP indicates variable importance in the projection; the two entries annotated as denatonium are distinct chromatographic features with different retention times and may represent different isobaric compounds; all annotations are putative (MSI level 2).

HMDB entries were available for 24 of the 45 differential metabolites. Among these, 9 metabolites were explicitly annotated as purely exogenous: epsilon caprolactam, dibutylamine, 3-methylbutyl angelate, pentaethylene glycol, imazapyr, butaxamine, (3R,4R)-7,12-dimethyl-3,4-dihydrobenzo(a)anthracene-3,4-diol, piperonyl butoxide, and bursopoietin. Another 12 metabolites were annotated with both endogenous and exogenous sources, and 3 metabolites had no source annotation.

Metabolic pathway enrichment analysis showed nominal enrichment (raw P < 0.05) in glycerophospholipid metabolism, valine, leucine and isoleucine biosynthesis and phenylalanine metabolism (**Figure 2D**, **Table 4**). None of these pathways survived FDR correction, and only one or two metabolites mapped to each pathway.

**Table 4:** Metabolic pathways between the PC and NC groups.

Path names	Total	Hits	Raw P	-log_10_(P)	FDR	Impact
Glycerophospholipid metabolism	36	2	0.007	2.130	0.623	0.0512
Valine, leucine and isoleucine biosynthesis	8	1	0.031	1.514	1.000	0
Phenylalanine metabolism	10	1	0.038	1.419	1.000	0.2381
Glycerolipid metabolism	16	1	0.060	1.219	1.000	0.10467
Sphingolipid metabolism	21	1	0.079	1.104	1.000	0.00406
Valine, leucine and isoleucine degradation	40	1	0.145	0.837	1.000	0.01084
Primary bile acid biosynthesis	46	1	0.166	0.781	1.000	0.03595

“Total” indicates the total number of metabolites in each pathway; “Hits” indicates the number of differential metabolites mapped to each pathway; “Impact” indicates pathway impact value.

### 3.3 ROC curve analysis and correlation with inflammatory markers

**Figure 3:**
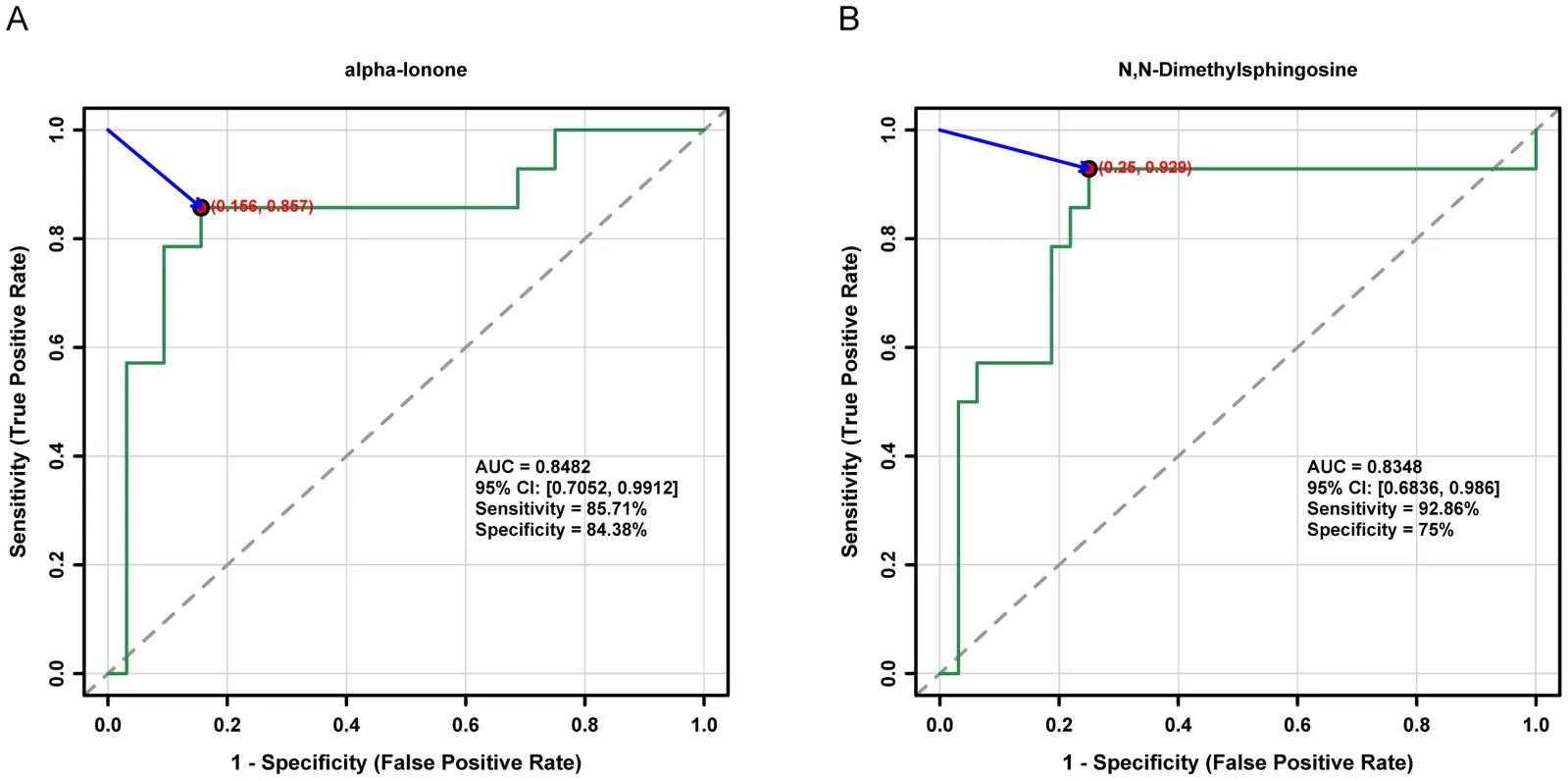
ROC curve analysis of alpha-ionone and N,N-dimethylsphingosine for distinguishing the PC and NC groups. **(A)** ROC curve for alpha-ionone. **(B)** ROC curve for N,N-dimethylsphingosine.

**Figure 4:**
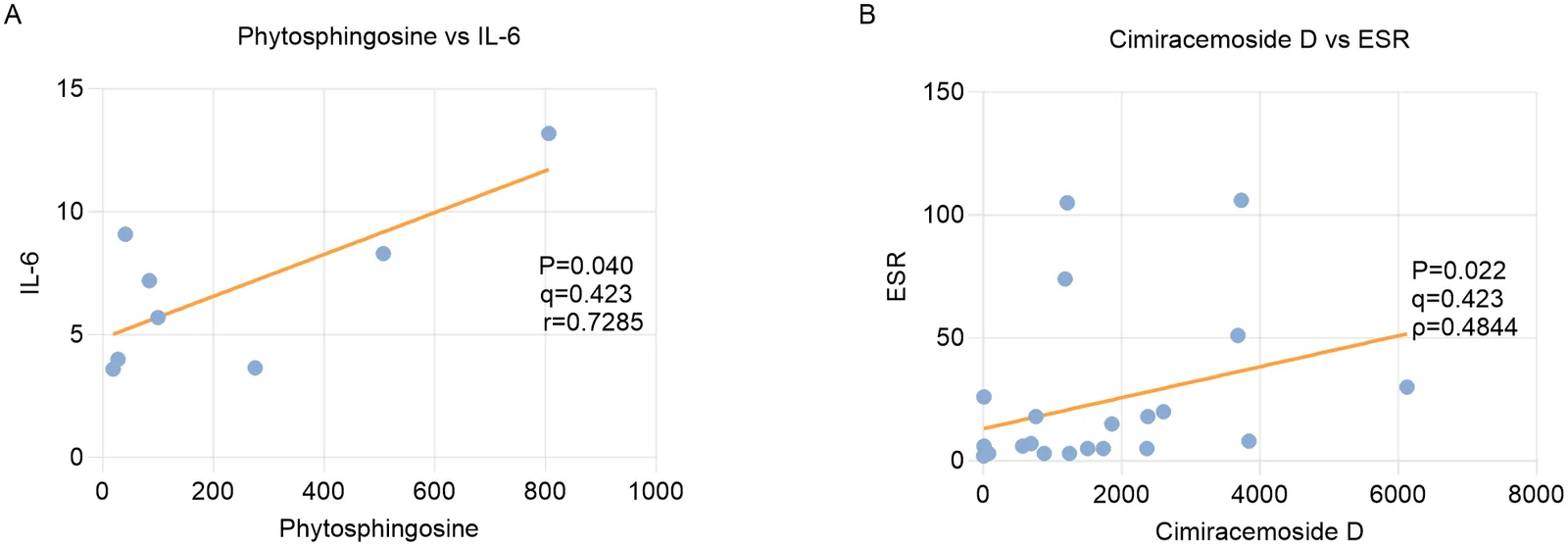
Correlation analysis between metabolites and inflammatory markers. **(A)** Correlation between phytosphingosine and IL-6 levels. **(B)** Correlation between cimiracemoside D and ESR. FDR-adjusted q = 0.423 for both correlations.

In this single-center discovery cohort without independent validation, ROC curve analysis revealed that alpha-ionone and N,N-dimethylsphingosine exhibited favorable preliminary discriminatory performance, with AUC values of 0.8482 and 0.8348, respectively (**Figure 3**). Specifically, alpha-ionone showed a sensitivity and specificity of 85.71% and 84.38%, respectively. In contrast, phenethylamine and phytosphingosine showed moderate preliminary discriminatory performance, with AUC values of 0.7679 and 0.7567, respectively. All four passed the FDR threshold (q < 0.05). Phytosphingosine correlated positively with IL-6 (Pearson’s r = 0.73, raw P = 0.040) (**Figure 4A**), but the association did not remain significant after FDR correction (q = 0.423), and we therefore treat it as exploratory.

### 3.4 Metabolic differences between PC and PA

**Figure 5:**
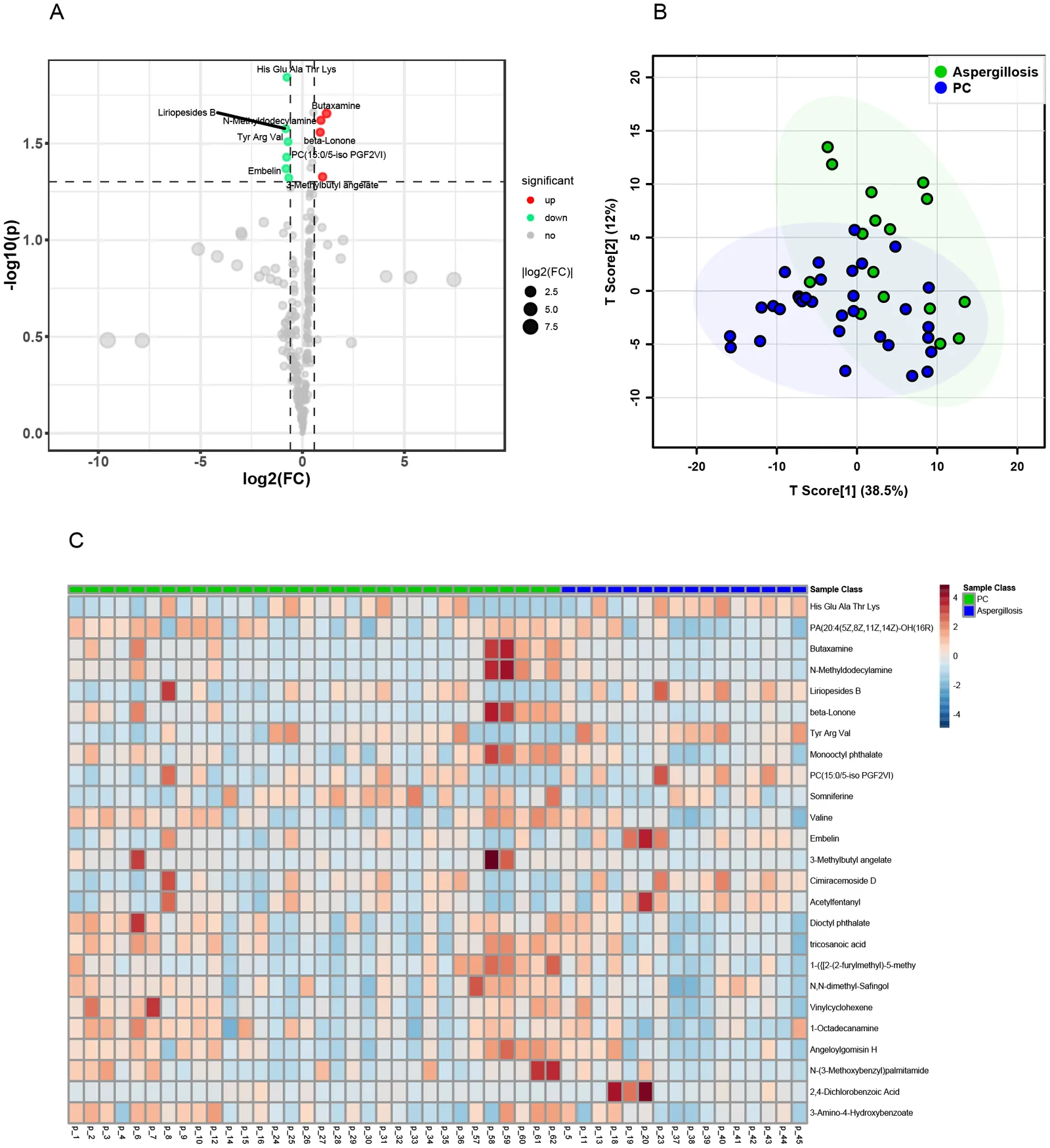
Metabolomic differences between the PC and PA groups. **(A)** Volcano plot illustrating differential metabolites between the two groups. **(B)** Partial least squares discriminant analysis (PLS-DA) score plot showing partial separation between the PC and PA groups. **(C)** Heatmap of selected metabolites showing differences in metabolic profiles between the two groups. Color gradients from blue to red indicate low to high relative metabolite abundance. PC, pulmonary cryptococcosis; PA, pulmonary aspergillosis.

A total of 268 metabolites were putatively annotated when comparing the PC and PA groups. The results of the univariate analysis were visualized using a volcano plot (**Figure 5A**). The PLS-DA results showed partial overlap between the two groups, although separation was still observed (**Figure 5B**), the heatmap is presented in **Figure 5C**, suggesting metabolic differences between these two types of pulmonary fungal infections.

Further screening identified eight differential metabolites: beta-ionone, butaxamine, embelin, Tyr-Arg-Val, His-Glu-Ala-Thr-Lys, cimiracemoside D, liriopesides B, and PC(15:0/5-iso PGF2VI). All eight metabolites reached significance at the nominal level (raw P < 0.05), but none remained significant after FDR correction (q = 0.535 for all eight metabolites). Pathway analysis pointed to inflammation-related metabolic pathways, including linoleic acid, alpha-linolenic acid, arachidonic acid, and glycerophospholipid metabolism (all raw P < 0.05); although none remained significant after FDR correction (q = 0.271–0.488). All four pathways were driven by a single putatively annotated metabolite, PC(15:0/5-iso PGF2VI), mapped only to a KEGG class entry. Among the differential lipids limited to metabolites classified as “Lipids and lipid-like molecules”, only beta-ionone and cimiracemoside D were shared between the PC vs NC comparison (16 lipids) and the PC vs PA comparison (4 lipids); the remaining 14 lipids were unique to PC vs NC, and 2 lipids were unique to PC vs PA.

### 3.5 Distribution characteristics of key metabolites

**Figure 6:**
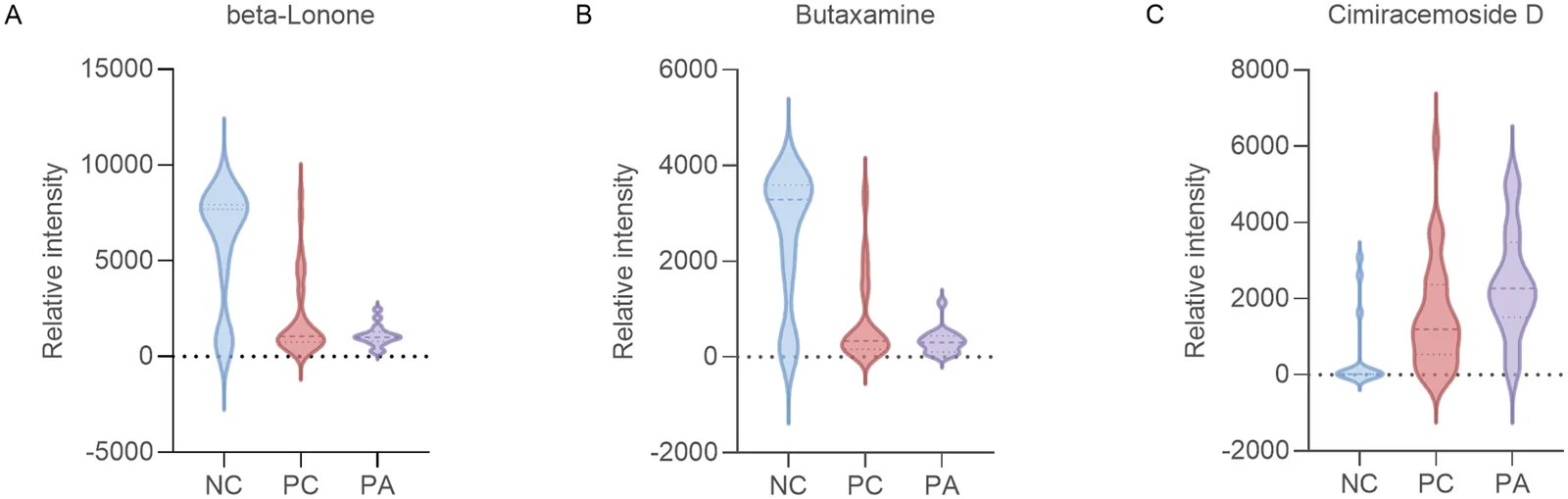
Distribution of relative expression levels of three candidate metabolites across different groups (violin plots). **(A)** beta-Ionone. **(B)** Butaxamine. **(C)** Cimiracemoside D. The width of the violin plot represents the density of the data distribution. Dashed lines indicate median and interquartile ranges. NC, healthy controls; PC, pulmonary cryptococcosis; PA, pulmonary aspergillosis.

Further analysis revealed that beta-ionone, butaxamine, and cimiracemoside D displayed a continuous gradient of changes across the NC, PC, and PA groups (**Figure 6**). Furthermore, cimiracemoside D was positively correlated with ESR levels (Spearman’s ρ = 0.48, raw P = 0.022) (**Figure 4B**), but this association likewise did not survive FDR correction (q = 0.423).

## 4 Discussion

In this study, untargeted metabolomic profiling of EBC was used to characterize the metabolic features of patients with PC and to explore disease-related metabolic alterations. Compared with traditional diagnostic methods, EBC collection is simple, noninvasive, and reproducible, facilitating the dynamic monitoring of disease progression. The results demonstrated a clear separation in the overall metabolic profiles between the PC and NC groups, suggesting that EBC may reflect metabolic changes in the local pulmonary microenvironment in response to infection.

EBC reflects the composition of airway lining fluid, which is continuously exposed to inhaled environmental constituents. The exogenous metabolites putatively annotated in this study originate from diverse sources: industrial chemicals (dibutylamine, epsilon caprolactam, pentaethylene glycol, decaethylene glycol, (3R, 4R)-7, 12-Dimethyl-3, 4-dihydrobenzo(a)anthracene-3, 4-diol), pesticide residues (imazapyr, piperonyl butoxide), botanical sources (3-methylbutyl angelate from Roman chamomile; cimiracemoside D from Cimicifuga racemosa), and synthetic bioactive compounds (butaxamine, denatonium). Their differential abundance between groups likely reflects variations in environmental exposure, lifestyle, and dietary patterns between patients and controls.

This finding underscores that EBC captures not only endogenous metabolic alterations but also exposome information, collectively constituting the metabolic profile of pulmonary cryptococcosis that effectively distinguishes disease states. The value of exogenous metabolites may arise from two explanations: first, disease-associated changes in pulmonary physiology such as altered epithelial permeability and mucociliary dysfunction may alter the deposition or clearance of inhaled xenobiotics; second, certain exogenous compounds possess biological activities that interact with host pathophysiological processes. The latter explanation is particularly relevant to alpha-ionone.

In this discovery cohort, ROC analysis revealed that alpha-ionone and N,N-dimethylsphingosine exhibited favorable preliminary discriminatory performance. Specifically, alpha-ionone showed a sensitivity of 85.71% and a specificity of 84.38% in distinguishing patients with PC from healthy individuals, indicating the presence of distinct metabolic alterations associated with PC. Notably, the sensitivity of alpha-ionone was numerically comparable to that of the serum CrAg test (87.5%, 21/24) and numerically superior to that of certain traditional etiological detection methods, pointing toward EBC-based metabolite detection as a promising candidate for complementary diagnosis that merits independent validation in larger cohorts. However, the observed comparison reflects only the statistical performance of different assays in this cohort and does not indicate equivalence in either mechanistic basis or clinical application. Alpha-ionone is widely present in carrots, raspberries, and other fruits and vegetables, as well as in plant essential oils (Aloum et al., 2020; Geng et al., 2023). The CCD1 enzyme required for alpha-ionone biosynthesis in plants is not expressed in humans (Baldermann et al., 2010); a previous review has indicated that mitochondrial BCO2 catalyzes asymmetric cleavage of beta-carotene to produce only beta-ionone (Aloum et al., 2020). Recent *in vitro* enzymatic studies have shown that although BCO2 can cleave alpha-carotene to putatively generate alpha-ionone, knockout mouse models suggest that this pathway exhibits extremely low metabolic efficiency *in vivo* (Bandara et al., 2025). Based on available evidence, alpha-ionone detected in EBC in this study most likely originates from exogenous intake, and the contribution of endogenous enzymatic synthesis is negligible.

Despite its exogenous origin, the downregulation of alpha-ionone in patients with PC may still be of pathophysiological relevance. Notably, this compound possesses both anti-inflammatory and antioxidant activities: in a UVB-irradiated keratinocyte model, it significantly alleviated oxidative stress and downregulated pro-inflammatory cytokine expression (Geng et al., 2023). Pathogens such as *Cryptococcus* can trigger host immune activation and reactive oxygen species generation (Vlasova-St Louis et al., 2021; Coelho et al., 2014), and reduced levels of such a bioactive exogenous compound in this pathological setting may indirectly reflect infection-related host oxidative stress responses. Alternatively, altered dietary intake patterns or changes in systemic clearance rates cannot be excluded, and further investigation is warranted.

3-methyl-2-oxovalerate appears in both isoleucine synthesis and degradation. In this study, the valine, leucine and isoleucine biosynthesis pathway was nominally enriched (raw P = 0.031), driven in part by this metabolite. In humans, this metabolite arises from isoleucine breakdown rather than *de novo* synthesis, since the body lacks the key upstream biosynthetic enzymes. Fungi such as *Cryptococcus* and *Aspergillus*, on the other hand, carry the full branched-chain amino acid synthesis machinery. Genetic studies have demonstrated that disruption of biosynthetic genes abolishes fungal pathogenicity: *ILV2* in *C. neoformans* is required for survival at 37 °C and full virulence (Kingsbury et al., 2004), *ilvC* in *A. fumigatus* is required for isoleucine and valine synthesis and virulence (Oliver et al., 2012), and *leuC* in *A. fumigatus* is required for leucine synthesis and virulence (Orasch et al., 2019). The nominal enrichment of the valine, leucine, and isoleucine biosynthesis pathway (raw P = 0.031, not significant following FDR correction), together with the elevation of 3-methyl-2-oxovalerate in patients with PC (FC = 2.2072), raises the possibility that pulmonary fungal metabolic activity may partly explain the observed changes. However, because this metabolite is a shared intermediate of both anabolic and catabolic pathways, the relative contributions of host degradation versus microbial biosynthesis cannot be distinguished based on the present data.

In this study, a substantial proportion of the differential metabolites were lipids or lipid-like molecules. Pathway analysis further implicated glycerophospholipid metabolism at a nominal significance level. Together, these findings suggest that lipid metabolic disorders may contribute to the pathogenesis and progression of PC. Sphingolipid molecules are not only essential components of cell membranes but are also closely involved in inflammatory responses, apoptosis, and immune signal transduction.

Alpha-Ionone and N,N-dimethylsphingosine identified in this study did not show significant differences between the PC and PA groups (P > 0.05). This finding suggests that these metabolites may primarily reflect host metabolic alterations associated with fungal infection rather than pathogen-specific characteristics, which may limit their ability to discriminate between different fungal pathogens.

In the present study, significant alterations were observed in metabolites such as N,N-dimethylsphingosine and phytosphingosine. Notably, phytosphingosine levels were positively correlated with IL-6, although this association did not survive FDR correction; even so, it leaves open the possibility that changes in sphingolipid metabolism may participate in the regulation of host inflammatory responses. Accumulating evidence indicates that sphingosine and its derivatives can modulate immune cell function and cytokine release (Quinville et al., 2021), thereby influencing the host immune response to pathogens. Therefore, alterations in sphingolipid metabolites in EBC may reflect changes in the local pulmonary immune microenvironment.

In contrast, although phytosphingosine and phenethylamine showed moderate preliminary discriminatory performance, their metabolic changes nonetheless generate the hypothesis that these metabolites are involved in *Cryptococcus* virulence-related processes. In the present study, we observed a significant decrease in the levels of phenethylamine, an intermediate in the phenylalanine metabolic pathway, in PC group. Melanin is a critical virulence factor in *Cryptococcus*, and cryptococcal laccases can utilize phenolic compounds to catalyze melanin synthesis (Brilhante et al., 2020). Given the close relationship between phenylalanine metabolism and the generation of such substrates, the decrease in phenethylamine may reflect increased activity of related metabolic pathways under infectious conditions. If so, this could support melanin synthesis and thereby affect *Cryptococcus* pathogenicity. Phytosphingosine may also be relevant to cryptococcal biology. In *Cryptococcus*, this sphingoid base can be converted into phytoceramides and inositol phosphorylceramides (IPCs), which are involved in plasma membrane homeostasis (Farnoud et al., 2014; Singh et al., 2017). Moreover, Glycoinositolphosphoryl ceramides (GIPCs), which contain a phytosphingosine backbone, differ in structure and abundance between encapsulated and acapsular *C. neoformans* strains (Heise et al., 2002), and the β1,2-xylosyltransferase Cxt1p functions in both capsule polysaccharide synthesis and glycosphingolipid xylosylation (Castle et al., 2008). Thus, the reduced phytosphingosine abundance observed in the PC group may be associated with cryptococcal membrane homeostasis and capsule-related glycan synthesis.

Although these mechanistic inferences are based primarily on metabolic pathway analyses and previous research findings, they only help make biological sense of the metabolic abnormalities observed in this study and remain speculative. Further functional experiments are required to validate these underlying mechanisms.

Through comparative analysis with PA, this study revealed that only a limited number of metabolites differed between the PC and PA groups at the nominal significance level. Differential metabolites were implicated in glycerophospholipid metabolism and polyunsaturated fatty acid metabolism, pathways closely linked to inflammatory mediators, oxidative stress, and immune regulation (Dyall et al., 2022). However, these enrichments arose from a single metabolite annotated to multiple KEGG pathway entries, and therefore do not indicate perturbation of four independent pathways. Concurrently, beta-ionone, butaxamine, and cimiracemoside D were consistently identified as differential metabolites in both comparisons involving the PC group, and displayed continuous gradient trends across the NC, PC, and PA groups. Furthermore, cimiracemoside D was positively correlated with ESR, although this did not remain significant after FDR correction. This distribution pattern suggests that these three metabolites may reflect variations in the intensity of the host inflammatory response rather than representing specific metabolic signatures. The consistent directional changes observed among these metabolites imply their potential involvement in shared host defense mechanisms. Among the three metabolites, the PA group showed the greatest alteration, suggesting that *Aspergillus* infection may induce stronger inflammation-associated metabolic changes than *Cryptococcus* infection.

Overall, the metabolic differences between PC and PA are more likely attributable to differences in the host inflammatory response. Integrating these findings with the pathway analysis raises the possibility that lipid metabolic alterations and PUFA-related pathway activity may contribute to a shared metabolic response during fungal infection. To some extent, this may contribute to the similarities observed in the metabolic profiles of the two diseases.

These observations cannot be directly compared with prior EBC metabolomics studies in pulmonary aspergillosis quantitatively. Previous studies used LC-HRMS to detect pathogen-specific secondary metabolites (Wei et al., 2022) or GC-MS to profile volatile terpenoids (Koo et al., 2014; Li et al., 2021), targeting metabolite classes that differ from those in the present study. We used a different LC-MS/MS system to profile the EBC metabolome, which revealed mainly host-related lipid and inflammatory alterations, as well as metabolites potentially associated with *Cryptococcus* virulence. Therefore, cross-study comparisons are limited to qualitative mechanistic interpretations, not quantitative diagnostic comparison.

Nevertheless, this study has several limitations. First, this was a single-center study with a relatively limited sample size, which may affect the robustness of the findings. The observed metabolic profiles may have been influenced by regional environmental exposures, dietary patterns, and local cryptococcal molecular types, limiting generalizability to other geographic regions. *Post-hoc* power analyses showed that the PC versus NC comparison had adequate power (≥0.80) for approximately 25 differential metabolites with large effect sizes, but fell below this threshold for the remaining 20 metabolites with moderate effects. The PC versus PA comparison lacked adequate power for all identified differential metabolites (power 0.52–0.72); similarly, pathway enrichment analyses and metabolite–inflammatory marker correlations were not significant after FDR correction. These findings should be considered exploratory, and the inability to identify pathogen-specific discriminative metabolites in the present study does not rule out real biological differences. Second, untargeted metabolomics provides broad coverage but lacks precise quantification of individual metabolites, and some metabolites require further validation. Third, pulmonary cryptococcosis is rare, so sample collection extended over a prolonged period. A small fraction of samples (8.1%) were stored at −80 °C for up to three years. We used snap-freezing, single-batch analysis and QC filtering to limit pre-analytical variation, but direct evidence for long-term EBC metabolite stability is still lacking. Some metabolites sensitive to prolonged storage may therefore have been missed. Additionally, no mechanistic experiments were performed, and the biological roles of the observed metabolic changes remain to be confirmed.

This study systematically analyzed the metabolic characteristics of EBC from patients with PC using UHPLC–QTOF–MS/MS. The putatively annotated alpha-ionone and N,N-dimethylsphingosine reflected distinct metabolic differences between patients with PC and healthy controls, while phytosphingosine and phenethylamine may have potential relevance to *Cryptococcus* virulence-related metabolism. Comparison with pulmonary aspergillosis further suggested that beta-ionone, butaxamine, and cimiracemoside D may reflect differences in the host inflammatory response rather than pathogen-specific metabolic signatures. These findings suggest that EBC-based metabolomic profiling characterized host inflammation-related and lipid metabolism-related alterations in PC, while its ability to achieve pathogen-specific discrimination remains limited.

To make EBC metabolomics a more reproducible and clinically informative tool, standardized pre-analytical workflows are essential. Multi-center studies with larger cohorts and harmonized sample collection and processing protocols will help determine whether EBC metabolic profiles can distinguish among different fungal lung infections. Targeted quantitative validation of the key differential metabolites, along with systematic assessment of their long-term stability at −80 °C, are important next steps.

## Data Availability

The original contributions presented in the study are included in the article/**Supplementary Material**. Further inquiries can be directed to the corresponding authors.
